# The Thiazole-5-Carboxamide GPS491 Inhibits HIV-1, Adenovirus, and Coronavirus Replication by Altering RNA Processing/Accumulation

**DOI:** 10.3390/v14010060

**Published:** 2021-12-30

**Authors:** Subha Dahal, Ran Cheng, Peter K. Cheung, Terek Been, Ramy Malty, Melissa Geng, Sarah Manianis, Lulzim Shkreta, Shahrazad Jahanshahi, Johanne Toutant, Rose Chan, Sean Park, Mark A. Brockman, Mohan Babu, Samira Mubareka, Karen Mossman, Arinjay Banerjee, Scott Gray-Owen, Martha Brown, Walid A. Houry, Benoit Chabot, David Grierson, Alan Cochrane

**Affiliations:** 1 Department of Molecular Genetics, University of Toronto, Toronto, ON M5S 1A8, Canada; subha.dahal@mail.utoronto.ca (S.D.); ran.cheng@mail.utoronto.ca (R.C.); terek.been@mail.mcgill.ca (T.B.); melissa.geng@mail.utoronto.ca (M.G.); sarah.manianis@mail.utoronto.ca (S.M.); Shahrazad.Jahanshahi@mail.utoronto.ca (S.J.); rose.chan@mail.utoronto.ca (R.C.); seonghungpark@mail.utoronto.ca (S.P.); scott.gray.owen@utoronto.ca (S.G.-O.); martha.brown@utoronto.ca (M.B.); 2 British Columbia Centre for Excellence in HIV/AIDS, Vancouver, BC V6Z 1Y6, Canada; pcheung@bccfe.ca (P.K.C.); mark.brockman@sfu.ca (M.A.B.); 3 Faculty of Health Sciences, Simon Fraser University, Burnaby, BC V5A 1S6, Canada; 4 Department of Biochemistry, University of Toronto, Toronto, ON M5S 1A8, Canada; ramy.malty@utoronto.ca (R.M.); walid.houry@utoronto.ca (W.A.H.); 5 Department of Microbiology and Infectious Diseases, Faculty of Medicine and Health Sciences, Université de Sherbrooke, Sherbrooke, QC J1H 5N4, Canada; Lulzim.Shkreta@usherbrooke.ca (L.S.); Johanne.toutant@usherbrooke.ca (J.T.); benoit.chabot@usherbrooke.ca (B.C.); 6 Research and Innovation Centre, Department of Biochemistry, University of Regina, Regina, SK S4S 0A2, Canada; mohan.babu@uregina.ca; 7 Department of Laboratory Medicine & Pathobiology, University of Toronto, Toronto, ON M5S 1A8, Canada; samira.mubareka@sunnybrook.ca; 8 Department of Medicine, McMaster University, Hamilton, ON L8N 3Z5, Canada; mossk@mcmaster.ca (K.M.); arinjay.banerjee@usask.ca (A.B.); 9 Department of Chemistry, University of Toronto, Toronto, ON M5S 3H6, Canada; 10 Faculty of Pharmaceutical Sciences, University of British Columbia, Vancouver, BC V6T 1Z3, Canada; david.grierson@ubc.ca

**Keywords:** HIV-1, adenovirus, coronavirus, RNA processing, inhibitor

## Abstract

Medicinal chemistry optimization of a previously described stilbene inhibitor of HIV-1, 5350150 (2-(2-(5-nitro-2-thienyl)vinyl)quinoline), led to the identification of the thiazole-5-carboxamide derivative (GPS491), which retained potent anti-HIV-1 activity with reduced toxicity. In this report, we demonstrate that the block of HIV-1 replication by GPS491 is accompanied by a drastic inhibition of viral gene expression (IC_50_ ~ 0.25 µM), and alterations in the production of unspliced, singly spliced, and multiply spliced HIV-1 RNAs. GPS491 also inhibited the replication of adenovirus and multiple coronaviruses. Low µM doses of GPS491 reduced adenovirus infectious yield ~1000 fold, altered virus early gene expression/viral E1A RNA processing, blocked viral DNA amplification, and inhibited late (hexon) gene expression. Loss of replication of multiple coronaviruses (229E, OC43, SARS-CoV2) upon GPS491 addition was associated with the inhibition of viral structural protein expression and the formation of virus particles. Consistent with the observed changes in viral RNA processing, GPS491 treatment induced selective alterations in the accumulation/phosphorylation/function of splicing regulatory SR proteins. Our study establishes that a compound that impacts the activity of cellular factors involved in RNA processing can prevent the replication of several viruses with minimal effect on cell viability.

## 1. Introduction

As of 2017, 75% of FDA-approved antivirals target discrete stages of the virus replication cycle (entry, genome replication, proteolytic cleavage of viral proteins) by inhibiting the activity of virus-encoded proteins [1]. Of the remainder, most are derivatives of interferons or target interactions with host cell surface receptors (i.e., maraviroc) [2]. Consequently, currently approved antivirals display effectiveness against only a limited range of viruses and can become ineffective by their selection for resistant viral strains. However, as obligate parasites, viruses are dependent upon a common subset of host cell processes for their replication [2,3]. Consequently, altering the ability of a virus to effectively use a host cell process may provide an alternative strategy to impede virus replication, generating a more robust barrier to virus resistance, and affect a broader spectrum of viruses with a single agent. Examples of such host-targeted therapeutic (HTT) strategies include the modulation of cell signaling, protein trafficking, lipid metabolism, epigenetic modifications, and RNA processing [2,3,4,5,6].

**Figure 1:**
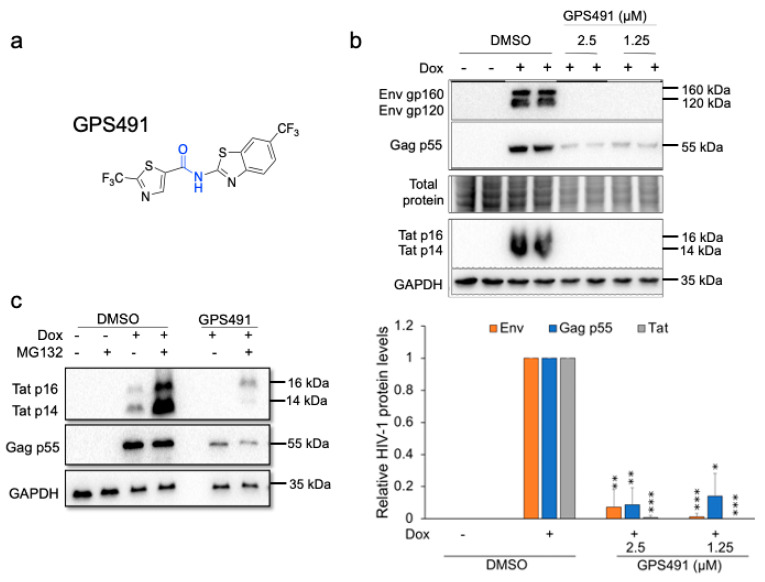
GPS491 inhibits expression of multiple HIV-1 proteins. (**a**) Chemical structure of GPS491. HeLa rtTA HIV∆mls cells were treated with either 2.5 µM or 1.25 µM GPS491. Cells grown with or without doxycycline (Dox) with 1% DMSO served as positive and negative controls, respectively. Cells were harvested for HIV-1 protein and RNA analyses after 24 h of induction. (**b**) Shown are representative Western blots showing the effects of compounds on HIV-1 Gag, Env, and Tat levels. Below is a summary of *n* = 3 independent experiments; data are indicated as mean ± SD; * *p* ≤ 0.05, ** *p* ≤ 0.01, and *** *p* ≤ 0.001. (**c**) Proteasome inhibition reverses the effect of GPS491 on Tat but not Gag expression. HeLa rtTA HIV∆mls cells were treated with DMSO or GPS491 +/− Dox for 24 h. Cells were treated with or without MG132 8 h prior to harvest, lysates were fractionated on 10% stain-free gels or 14% SDS PAGE gels, and the resulting blots were probed for HIV-1 Gag or Tat, respectively. Band intensity was quantified relative to Dox-induced DMSO control and normalized to total protein load (Stainfree) for Gag blot or normalized to GAPDH for Tat blot using Bio-Rad ImageLab software.

Previously, our group demonstrated that the stilbene 2-(2-(5-nitro-2-thienyl)vinyl)quinoline (designated 5350150) inhibits HIV-1 replication by altering viral RNA accumulation [7]. However, the limited therapeutic index (CC_50_/EC_50_ ratio) of 5350150 indicated that further optimization was necessary for its application as an antiviral against HIV-1. Synthesis and evaluation of a library of di(hetero)aryl amide analogs of 5350150 led to the selection of the thiazole carboxamide compound GPS491 [8] (Figure 1a, Table 1) for further study as an antiviral agent. In this report, we determine that, similar to 5350150, GPS491 blocks HIV-1 protein expression in part due to changes in viral RNA accumulation and processing. Additional tests determined that GPS491 also inhibits the replication of human adenovirus type 5 and several coronaviruses, demonstrating pan-antiviral activity of the compound. In the case of adenovirus, GPS491 addition altered E1A RNA processing and inhibited expression of other viral early genes resulting in the loss of adenovirus DNA replication and late viral gene expression. Consistent with the observed changes in viral RNA processing, treatment with GPS491 induced selective changes in SR protein accumulation/modification as well as function with minimal cytotoxic effects. Together, these findings support the hypothesis that compounds affecting viral RNA processing/expression can be used to suppress the growth of different viruses.

## 2. Materials and Methods

### 2.1. Cell Lines and Virus Strains

Effects of compound treatment on HIV-1 gene expression were initially assessed in the context of HeLa cells stably transduced with an inducible Tet-On HIV-1 system, as previously described [9,10,11]. The provirus was modified by either deletion in the reverse transcriptase and integrase region of the pol gene by an *Mls*I restriction digest (HeLa rtTA HIV∆*Mls* cell line) or GFP fusion to Gag, deleting the PR- and RT-coding regions (HeLa rtTA HIVGagGFP cell line). Tat and its TAR binding sites were inactivated so that HIV-1 gene expression was only induced in the presence of doxycycline (Dox). HeLa cells containing HIV-1 provirus and J-Lat 10.6 [12] cells were maintained in Iscove’s Modified Dulbecco’s Medium (IMDM), and Roswell Park Memorial Institute (RPMI) 1640 Medium (Wisent Corp., Saint-Jean Baptiste, QC, Canada), respectively, supplemented with 10% (*v*/*v*) fetal bovine serum (FBS, Wisent Corp., Saint-Jean Baptiste, QC, Canada), 1% penicillin/streptomycin (P/S, Wisent Corp., Saint-Jean Baptiste, QC, Canada), and 0.2% Amphotericin B (Wisent Corp., Saint-Jean Baptiste, QC, Canada). The indicator cell line CEM-GXR was maintained and used as described previously [13]. A549 cells (human lung carcinoma) were obtained from the American Type Culture Collection (ATCC, Manassas, VA, USA) at passage level 76 and used between passages 89 and 110. HEK 293 (human embryonic kidney) cells were obtained from F. Graham, McMaster University, Hamilton, Ontario, Canada, at passage 24 and were used between passages 58 and 90. A549 and HEK 293 cells were cultured in Eagle’s minimum essential medium (MEM, Gibco, Waltham, MA, USA) supplemented with 10% FBS (Sigma-Aldrich, St. Louis, MO, USA) plus penicillin (100 U/mL) and streptomycin (100 μg/mL). Huh7 cells for infection with coronaviruses were maintained in Dulbecco’s Modified Eagle Medium (DMEM, Wisent Corp., Saint-Jean Baptiste, QC, Canada) with high glucose (HyClone) supplemented with 10% heat-inactivated FBS and 1% P/S. All the cell lines were maintained at 37 °C in a humidified incubator with 5% CO_2_. HIV-1 reverse transcriptase-resistant virus (E00443) was obtained from Dr. Zabrina Brumme (Simon Fraser University, Burnaby, BC, Canada). HIV-1 clade A (97USSN54 (N54)), HIV-1 clade B (IIIB), integrase inhibitor-resistant virus (11845), and protease inhibitor-resistant virus (2948) were obtained through the NIH AIDS Research and Reference Reagent Program, Division of AIDS, NIAID, NIH. Adenovirus used in the studies was HAdV-C5. Coronaviruses 229E and OC43 were obtained from ATCC. SARS-CoV2 used is the SB2 strain was obtained from Dr. S. Mubareka.

### 2.2. GPS491 Inhibition of Virus Replication

The compound was prepared as outlined in Zamiri, M., et al. [8] and dissolved to 10 mM or 1 mM stock concentration in dimethyl sulfoxide (DMSO) and stored at −20 °C for subsequent experiments. Compound purity was determined to be >95% as assessed by high-resolution mass spectrometry. For assays involving cell lines containing the integrated HIV-1 provirus, cells were seeded at a confluence of 70% on 6-well plates in IMDM or RPMI 1640 complete medium and treated with GPS491 at the desired concentration for 24 h. Control wells contained DMSO at 1% final concentration. Necessary cells were induced with Dox or prostratin at a final concentration of 2 μg/mL and 1 µg/mL, respectively, at the same time the compound was added. Twenty-four hours post compound treatment, cells were harvested in 2 mM EDTA and 1×PBS for Western blotting and RNA analyses.

To assess the activity of compounds against HIV-1 (WT or strains resistant to various antiretroviral drugs), assays were performed in the context of CEM-GXR cells, an immortalized CD4^+^ T-lymphocyte line that expresses GFP due to the activation of an integrated Tat-dependent promoter upon HIV-1 infection [13]. HIV-1 spread in culture was monitored using flow cytometry. CEM-GXR cells naturally express the HIV-1 receptor CD4 and coreceptor CXCR4 and have been engineered to express HIV-1′s other coreceptor, CCR5. Cultures were infected with different HIV-1 strains of interest, differing in their subtypes (A and B), and resistance to four major drug targets (reverse transcriptase, protease, integrase, and coreceptor antagonist). The HIV-1 inhibition assay was performed in a 96-well plate, where CEM-GXR cells were inoculated via co-culture with CEM-GXR cells infected with different viral strains of interest (1%, initial proportion of cells). GPS491 was then immediately added to the inoculated cultures such that the final compound concentrations were between 100 nM and 10 μM. The percentage of infected cells in the culture was determined by flow cytometry on day 3 p.i. The inhibition curves were fitted by nonlinear regression using GraphPad Prism software (San Diego, CA, USA), allowing for EC_50_ calculation. All data are reported as the mean ± s.e.m. from three independent determinations. The cytotoxicity of GPS491 was investigated by exposing uninfected CEM-GXR cells to GPS491 and assessing viability using the Guava ViaCount flow cytometry assay according to the manufacturer’s instructions (Luminex Corp., Toronto, ON, Canada). In brief, CEM-GXR cells were seeded into 96-well plates at 8 × 10^4^ cells/well in the absence or presence of GPS491 at the indicated concentrations. Three days later, 25 μL of cell suspension was mixed with 225 μL of Guava ViaCount reagent, the mixture was incubated for 5 min at room temperature, and then analyzed on a Guava easyCyte 8HT flow cytometer (Luminex Corp., Toronto, ON, Canada). Data analysis was performed using the EasyFit analysis feature in the ViaCount software module in GuavaSoft v2.7 (Luminex Corp., Toronto, ON, Canada).

Studies involving peripheral blood mononuclear cells (PBMCs) were performed as described by Mwimanzi et al. [14]. Briefly, PBMCs from three HIV-1-negative donors were activated for 3 days using 5 μg/mL phytohemagglutinin in RPMI media supplemented with 10% heat-inactivated FBS (R10+), infected with HIV-1_IIIB_ or HIV-1_BaL_ for 6 h, washed and resuspended in R10+ media supplemented with 100 U/mL IL-2 (Sigma-Aldrich, St. Louis, MO, USA), seeded into 96-well U-bottom plates at 150,000 cells/well, and treated with GPS491 or 0.1% DMSO. On day 4 and day 7 p.i., the culture media, compound, and IL-2 were replenished. Supernatant p24^Gag^ levels were quantified on day 11 p.i. by ELISA (Xpress Bio, Frederick, MD, USA) as per manufacturer’s instructions. To quantify cytotoxicity of the compound on PBMCs, the above procedure was repeated with uninfected PBMCs from the same donors. On day 11 post treatment, culture viability was assessed using Guava ViaCount reagent (Luminex Corp., Toronto, ON, Canada) and flow cytometry, as per the manufacturer’s instructions.

To examine the effect of GPS491 on adenovirus replication, A549 cells were seeded in a 6-well plate at a density of 5 × 10^5^ cells/well. Cells were infected one day post seeding at an input multiplicity of infection (MOI) of 100 IU/cell of human adenovirus serotype 5 (HAdV-C5). After 1 h of adsorption at 37 °C, inoculum was removed and replaced with a fresh culture medium containing 1% DMSO (solvent control) or compound dissolved in DMSO (duplicate wells per condition). Progeny virus was harvested 24 h p.i. by scraping the cells into the culture fluid, followed by freeze-thaw of suspension five times with vortexing. The lysate was clarified by centrifugation at 500× *g* for 5 min and titrated by endpoint dilution in HEK 293 cells [15].

To examine the dose-dependent effects of GPS491 on coronavirus replication, Huh7 cells were seeded in a 96-well plate and infected at an input MOI of 0.1 and 1 for 229E and OC43, respectively. To study the effects of the compound on viral protein levels and viral RNA in media (sups), cells were seeded in a 6-well plate and infected with 229E, OC43, or SARS-CoV2 [16] at an input MOI of 0.03, 0.3, and 1, respectively. Infection with virus inoculum was done in serum-free DMEM for an hour with gentle rocking of the plate back and forth every 20 min during the course of infection. Virus inoculum was subsequently removed after an hour, cells washed with 1× PBS, and treated with DMSO or a compound diluted in DMEM supplemented with 2% FBS and 1% P/S. Cell media were harvested at 2 d p.i. for 229E or SARS-CoV2 infection and at 4 d p.i. for OC43 infection, followed by heat inactivation at 95 °C for 5 min. For time of addition studies, cells were infected at an input MOI of 2, DMSO or GPS491 added at various times post virus removal, and media harvested at 24 h post infection to assay viral RNA levels. Media were directly used to detect viral RNA using Luna^®^ Universal One-Step RT-qPCR Kit (New England Biolabs, Ipswich, MA, USA) as per the manufacturer’s instructions. Each reaction was set up as follows in qPCR 384-well plate: 5 µL Luna Universal One-Step Reaction Mix (2×), 0.5 µL Luna WarmStart^®^ RT Enzyme (20×), 0.2 µL of each 5′ and 3′ primers (10 µM), and 1 µL of media (template RNA) in a total reaction volume of 10 µL. Sequences used to detect coronavirus RNA in media are provided in Supplementary Table S1. The qPCR cycling conditions were as follows: reverse transcription at 55 °C for 10 min and initial denaturation at 95 °C for 1 min, followed by 40 cycles of denaturation at 95 °C for 10 s and extension at 60 °C for 30 s. The melting curve protocol followed with 15 s at 95 °C and then 15 s each at 0.2 °C increments between 60 °C and 95 °C. Melting and standard curves were generated by the CFX Maestro Software (version 1.1, Bio-Rad, Mississauga, ON, Canada).

### 2.3. Effect on Viral and Host Protein Expression

To assess the effects on viral proteins by Western blot, cell extracts were prepared in RIPA buffer (50 mM Tris-HCl pH 7.5, 150 mM NaCl, 1% NP-40, 0.5% sodium deoxycholate, 0.1% SDS) and fractionated on 10% TGX acrylamide stain-free (Bio-Rad, Mississauga, ON, Canada) or 14% SDS-PAGE gels. Stain-free gels were directly imaged on ChemiDoc MP Imager (Bio-Rad) to measure total protein levels, which served as a loading control. Following imaging for total protein levels, protein was transferred to PVDF using the Trans-Blot Turbo Transfer System (Bio-Rad, Mississauga, ON, Canada). Blots were blocked in either 5% Milk-TBS-T (5% Milk, 0.05% Tween-20, 1× TBS) or 3% BSA-TBS-T (3% BSA, 0.05% Tween-20, 1× TBS) prior to incubating with primary antibodies (all diluted in either 5% BSA-TBS-T or 5% Milk-TBS-T). Primary antibodies used to evaluate the effect of GPS491 on HIV-1 protein levels were purified mouse anti-p24 supernatant from anti-HIV-1 Gag hybridoma 183, mouse anti-gp120 hybridoma 902, mouse α-Tat hybridoma (1D9) (all obtained from NIH AIDS Reagent Program), and rabbit α-GAPDH (Sigma-Aldrich, St. Louis, MO, USA). To evaluate the effects of GPS491 on SR protein expression levels, blots were probed with one of the following antibodies; mouse α-SRSF1 (Life Technologies, Waltham, MA, USA Cat: 32-4500), rabbit α-SRSF2 (BD Pharmingen, San Diego, CA, USA, Cat: 556363), mouse α-SRSF3 (Life Technologies, Waltham, MA, USA, Cat: 33-4200), rabbit α-SRSF4 (Novus Biologicals, Toronto, ON, Canada, Cat: NBP2-04144), rabbit α-SRSF5 (MBL, Woburn, MA, USA RN082PW), rabbit α-SRSF6 (Novus Biologicals, Toronto, ON, Canada, Cat: NBP2-04142), rabbit α-SRSF7 (Abcam, Cambridge, UK, Cat: ab137247), rabbit α-SRSF9 (MBL, Woburn, MA, USA, Cat: RN081PW), mouse α-SRSF10 (Novus Biologicals, Toronto, ON, Canada, Cat: H00010772-M07), rabbit α-Tra2β (Abcam, Cambridge, UK, Cat: ab31353), mouse α-CLK1 (Santa Cruz, Biotechnology Inc., Dallas, TX, USA Cat: 515897), rabbit α-CLK2 (Abcam, Cambridge, UK, Cat: ab65082), mouse α-CLK3 (Abnova, Walnut, CA, USA Cat: H0001198-M05), and rabbit α-SRPK1 (Cedarlane, Burlington, ON, Canada Cat: OAAN01583). Adenovirus E1A and hexon proteins were detected using rabbit α-E1A (Santa Cruz Biotechnology Inc., Dallas, TX, USA Cat: sc-430) and undiluted hybridoma (2Hx-2) media, respectively, as previously detailed [17]. To measure the effect of the compound on 229E, OC43, or SARS CoV-2 nucleocapsid (N) protein expression, either mouse α-coronavirus antibody clone FIPV3-70 (Novus Biologicals, Toronto, ON, Canada Cat: NB10064754), mouse α-coronavirus group antigen antibody clone 542-7D (Millipore Sigma, Burlington, MA, USA, Cat: MAB9013), or rabbit anti-SARS CoV2 nucleocapsid antibody (SinoBiological, Beijing, China, Cat: 40143-R019) was used, respectively. Changes in OC43 spike protein expression were assessed using mouse monoclonal antibody 541-8F (Sigma-Aldrich, St. Louis, MO, USA, Cat: MAB9012). Following overnight incubation with primary antibodies at 4°C, blots were washed and incubated in appropriate secondary antibody (either α-mouse or α-rabbit horseradish peroxidase (HRP)-conjugated IgG (Jackson ImmunoResearch, West Grove PA, USA) for an hour at room temperature. After subsequent washes, signals were detected by ECL/ECL Plus (Perkin-Elmer, Waltham, MA, USA), or Clarity Western ECL reagent (BioRad, Mississauga, ON, Canada) and imaged using a ChemiDoc MP Imager (Bio-Rad, Mississauga, ON, Canada). To test whether changes in protein migration were attributable to altered phosphorylation, extracts were treated with lambda phosphatase (New England Biolabs, Ipswich, MA, USA Cat: P0753S) prior to running on gels. Relative intensity of detected bands was normalized to corresponding bands of the loading control (GAPDH or total protein using Bio-Rad ImageLab software, Mississauga, ON, Canada).

To examine the effects of GPS491 on adenoviral protein (hexon) expression by immunofluorescence, cells were fixed at 24 h p.i. with 3.7% PFA for 15 min, washed with 1× PBS, and permeabilized for 15 min with 0.1% Triton X-100 in PBS (PBT), followed by blocking for 45 min with 5% BSA in PBT (BSA-PBT). Cells were then incubated in primary antibody (undiluted culture fluid from 2HX-2 hybridoma cells to probe for hexon) for 45 min followed by three washes with 1× PBS and incubation in secondary antibody (goat anti-mouse labeled with AlexaFluor488 (Jackson ImmunoResearch, West Grove, PA, USA) at 1:200 dilution) for 45 min. After subsequent washes in PBT and PBS, coverslips were mounted on a glass slide with PBS containing 0.25 µg/mL DAPI. Cells were viewed with 10× objective using a Leica DMR microscope and images captured with OpenLab imaging software version 2.0.7 (Santa Clara, CA, USA).

### 2.4. Toxicity Assays

Cytotoxicity of GPS491 was assessed by using a CellTiter-Glo luminescent cell viability assay (Promega, Madison, WI, USA), alamarBlue or Trypan blue exclusion (Life Technologies, Waltham, MA, USA) and expressed relative to cells treated with DMSO (1%) alone. alamarBlue was added to culture medium prior to harvest/fixation, cells were incubated at 37 °C in a 5% CO_2_ humidified incubator for 2–6 h, and fluorescence reflecting cell metabolic rate was measured using Bio Tek Cytation 5 (Winooski, VT, USA or TECAN infinite 200Pro fluorescence plate reader (Meilen, Zurich, Switzerland) (fluorescence detection, 560 nm (ex)/590 nm (em)).

### 2.5. RNA/DNA Analysis

Total RNA was extracted using the BioRad Aurum Total RNA Lysis Kit (BioRad, Mississauga, ON, Canada) as per the manufacturer’s instructions. For HIV-1 samples, purified RNA (0.5–2 µg) was reverse transcribed using M-MLV (Invitrogen) to generate complementary DNA (cDNA). HIV-1 and β-actin mRNA levels in DMSO- or compound-treated samples were quantified by qPCR as described [9]. Target quantification was evaluated using the absolute quantification method, normalized to β-actin expression, and expressed relative to DMSO treatment. Adenovirus cDNA was produced from extracted RNA by reverse transcription (RT) with the Bio-Rad iScript cDNA Synthesis Kit, according to the manufacturer’s protocol, using 1 µg of RNA in a reaction volume of 20 µL. The conditions were as follows: 5 min at 25 °C, 20 min at 46 °C, and 1 min at 95 °C. Subsequent qPCR analysis of the cDNA, diluted 1/100, was done in technical triplicates using Bio-Rad SsoAdvanced Universal SYBR Green Supermix with primers (Supplementary Table S1) at 500 nM final concentration, according to the manufacturer’s protocol. Cycling parameters were 98 °C for 3 min, then 40 cycles of 95 °C for 15 s, and 60 °C for 30 s. The abundance of adenovirus RNA in treated samples, relative to TBP RNA (control), was normalized to RNA abundance at 24 h p.i. in 0.1% DMSO.

The effect of compound treatment on splice site selection within the HIV-1 MS RNA class was analyzed by RT-PCR as described previously [18]. Amplicons were resolved on 7% polyacrylamide gels (1× TBE) and signals detected using ethidium bromide staining in a BioRad ChemiDoc imager (Mississauga, ON, Canada). Analysis of images was performed using ImageLab to calculate levels of HIV-1 MS mRNA isoforms, measured as signal intensity of an individual isoform divided by the total signal of all visible viral RNA species in a sample. Analysis of E1A RNA splicing was performed as previously described [17].

For adenovirus DNA analysis, cells were seeded on 6-well plates at a density of 5 ×10^5^ cells per well. The next day, cells were infected with HAdV-C5 (input MOI 100 IU/cell) for 1 h, and inoculum was removed and replaced with a culture medium containing DMSO or GPS491. At the indicated times p.i., the culture fluid was removed, and cells were lysed in situ with lysis buffer supplied with Norgen Biotek DNA extraction kit (Winooski, VT, USA, Cat: 53100). Adenovirus DNA levels were determined using the E2B primer pair (see Supplementary Table S1). Experiments were done using biological duplicates, each measured in technical triplicates. Reactions contained 6 μL of Bio-Rad SsoAdvanced Universal SYBR Green Supermix, 0.5 μL reverse primer (16.5 μM), 0.5 μL forward primer (16.5 μM), and 5 μL of the DNA sample diluted 1/10 in sterile distilled water. Cycling parameters were as follows: 95 °C for 30 s, and 40 cycles of 95 °C for 15 s and 60 °C for 30 s (as detailed in Bio-Rad SsoAdvanced Universal SYBR Green Supermix user manual). Data were analyzed using Bio-Rad Maestro software (Mississauga, ON, Canada).

### 2.6. Role of Proteasome in Reducing HIV-1 Tat Expression

To determine whether the effect of GPS491 on HIV-1 Tat expression could be reversed by inhibition of the proteasome, a compound treatment assay was performed as previously described with the addition of 10 µM MG132 (Sigma-Aldrich, St. Louis, MO, USA) to DMSO/compound-treated cells 8 h prior to harvesting [19]. Lysates were fractionated on 10% TGX stain-free (Bio-Rad, Mississauga, ON, Canada) or 14% SDS-PAGE gels, and blotted and probed with antibodies for Gag, Tat, or GAPDH in at least three independent experiments.

### 2.7. Statistical Analysis

Data are representative of at least three independent experiments, each performed in duplicate, and values are represented as mean ± standard deviation unless otherwise stated. Statistical significance comparisons between two samples were calculated using the paired two-tailed student’s t test (Microsoft Excel).

## 3. Results

### 3.1. GPS491 Inhibits HIV-1 Replication by Disrupting Viral RNA Accumulation

To assess the HIV-1 inhibitory activity of GPS491, we initially examined its ability to suppress replication of multiple strains of the virus in the CEM-GXR cell line that expresses GFP upon HIV-1 infection [13]. As summarized in Table 1 (see Supplementary Figure S1 for titration data), treatment of HIV-1-infected CEM-GXR cultures with nanomolar doses of GPS491 suppressed replication of all HIV-1 strains tested including clade A and B wild-type HIV-1 strains as well as variants resistant to different antiretroviral drugs (reverse transcriptase, protease, integrase, or entry inhibitors) (EC_50_~250 nM, CC_50_ = 12,052 nM). Inhibition of HIV-1 replication was also observed in the context of human PBMCs (Table 1). Taken together, these data suggest that GPS491 may be used in conjunction with other combination antiretroviral therapy (cART) regimens to enhance the control of HIV-1 infection.

To gain insight into its mechanism of action, we assessed the effect of GPS491 addition on HIV-1 gene expression using a HeLa cell line (HeLa rtTA HIV∆mls) transduced with a doxycycline-inducible HIV-1 provirus [9,10,11]. Western blots revealed >90% reduction in the accumulation of all HIV-1 proteins examined (Gag, Env, Tat) (Figure 1b) at low µM doses of GPS491. Parallel studies revealed a similar pattern of altered HIV-1 protein expression in the context of the J-Lat 10.6 cell line (a Jurkat CD4+ T cell line infected with a replication defective HIV-1 provirus expressing GFP from the position of Nef [12]), with a reduction of both Gag and GFP synthesis at low µM doses of GPS491 (see Supplementary Figure S2).

**Figure 2:**
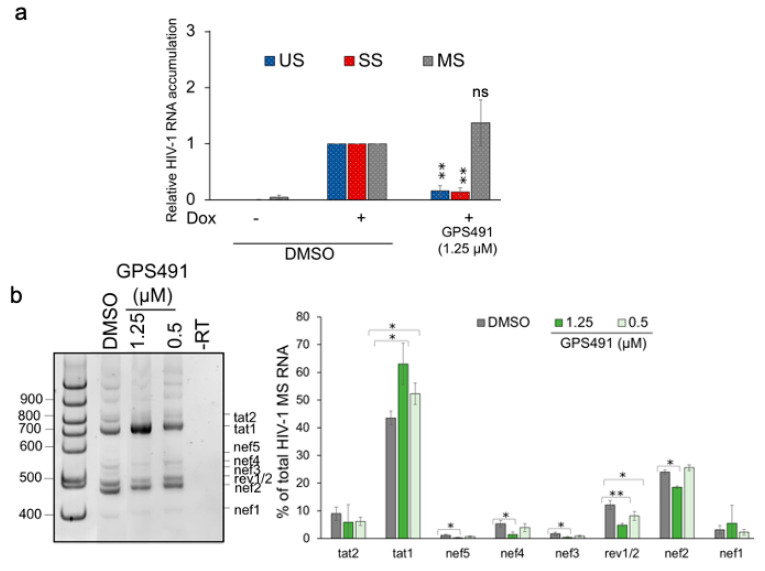
Effects of GPS491 on HIV-1 RNA accumulation and processing. HeLa rtTA HIV∆mls cells were treated with DMSO or varying concentrations of GPS491 +/− doxycycline (Dox) for 24 h. Cells were then harvested, total RNA was extracted, and cDNA was generated. Samples were subsequently analyzed (**a**) by RT-qPCR to measure levels of HIV-1 US, SS, and MS RNA levels or (**b**) by RT-PCR to measure levels of different MS RNA isoforms. Shown is a representative gel and summary quantitation of results from *n* > 3 independent assays. Data are indicated as mean ± SD, * *p* ≤ 0.05, ** *p* ≤ 0.01, ns—not significant.

To understand the basis for the observed alterations in viral protein expression, we examined the effect of GPS491 on HIV-1 RNA accumulation. In the case of HIV-1, the 9 kb primary viral transcript is alternatively spliced into ~69 mRNAs to express nine encoded viral proteins [20,21,22]. The viral mRNAs are classified into three groups: unspliced (US, encoding Gag and Gagpol), singly spliced (SS, used to make Vif, Vpr, Vpu, Tat p14, or Env), or multiply spliced (MS, encoding Tat p16, Rev, or Nef) RNAs. RT-qPCR analysis revealed that treatment with GPS491 reduced HIV-1 US and SS viral RNA accumulation while slightly increasing MS RNA levels (Figure 2a). The reduced accumulation of HIV-1 US and SS RNAs accounts for the loss of Gag, Env, and Tat p14, but the loss of Tat p16 was unexpected given the significant levels of MS RNA present. One possibility was that GPS491 induced an alteration in splice site usage in the MS class of viral RNAs, similar to the related compound 5350150 [7]. RT-PCR analysis [18] of the HIV-1 MS RNAs (Figure 2b) determined that a dose of GPS491 (1.25 µM), which reduced HIV-1 protein expression, shifted the relative abundance of MS RNAs, increasing the abundance of Tat1 mRNA while decreasing levels of Rev1/2 mRNAs. MS RNA relative abundance was similar to the DMSO control at a lower dose of GPS491 (0.5 µM). With GPS491 addition increasing the levels of Tat mRNA, the basis for the loss of Tat p16 protein (encoded by Tat1 mRNA) was not immediately clear. An alternative explanation for the loss of Tat protein expression is that GPS491 induced changes in the rate of Tat synthesis and/or degradation as seen previously with other modulators of HIV-1 replication [19,23]. To test this hypothesis, we examined whether treatment with the proteasome inhibitor MG132 could restore Tat protein expression. As shown in Figure 1c, addition of MG132 to cultures 8 h prior to harvest resulted in a significant increase in Tat (both p16 and p14, expressed from MS and SS viral RNAs, respectively) in the presence of DMSO as well as expression of predominantly p16 Tat in the presence of GPS491. Although expression of Tat p16 was increased with MG132 and GPS491 addition, there was no detectable change in Gag levels consistent with the reduced level of its mRNA.

### 3.2. GPS491 Inhibits Adenovirus Replication

**Figure 3:**
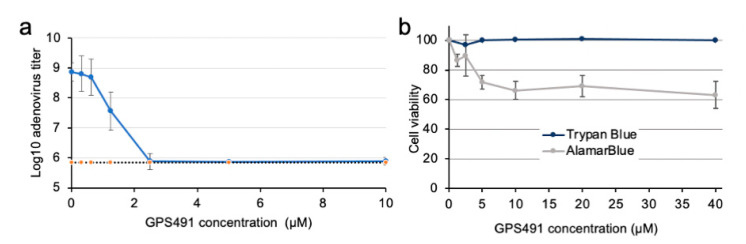
GPS491 inhibits adenovirus (HAdV-C5) replication in A549 cells. (**a**) A549 cells were uninfected or infected with human adenovirus serotype 5 (HAdV-C5) at an input MOI of 100 IU/cell for 1 h after which the virus inoculum was removed and replaced with media containing DMSO or the indicated concentrations of GPS491. After 24 h, the virus was harvested by scraping cells into the culture fluid and the cell suspension was collected for titration of infectious virus by endpoint dilution (blue line). T0 (dotted line, orange dots) is a sample harvested immediately after the 1 h adsorption period to measure the residual virus from the inoculum. Error bars represent the standard deviation of three independent experiments. (**b**) A549 cells were treated with the indicated doses of GPS491 for 24 h and then assessed for changes in metabolic activity by alamarBlue (grey line) or for viability by staining with trypan blue (blue line).

**Figure 4:**
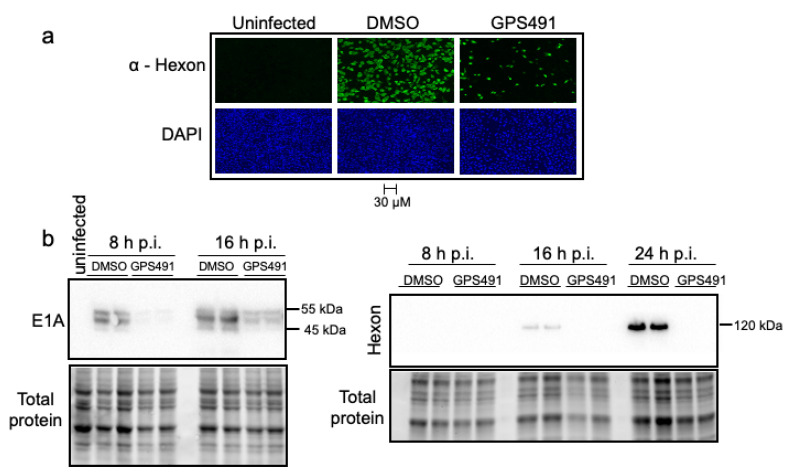
GPS491 delays E1A production and inhibits adenovirus hexon expression. A549 cells were infected with HAdV-C5 at an input MOI of 100 IU/cell for 1 h after which the virus inoculum was removed and replaced with media containing DMSO or GPS491 (2.5 µM). (**a**) Cells were fixed 24 h after infection and hexon expression assessed by immunofluorescence. Scale is the same for all images. (**b**) Cells were harvested 8 h, 16 h, or 24 h p.i. and lysates were fractionated on SDS-PAGE gels. Shown are the representative Western blots showing the expression levels of E1A and hexon proteins in the control versus treated lysates.

The ability of GPS491 to induce changes in HIV-1 RNA accumulation that correlate with inhibition of virus replication suggested that we examine whether GPS491 affected replication of adenovirus, another virus with a similar dependence on the host RNA processing machinery. As shown in Figure 3a, addition of GPS491 to cultures 1 h after HAdV-C5 virus inoculation reduced adenovirus yield ~1000-fold with an IC_50_ of ~1 µM. Analysis of cells following GPS491 treatment in A549 cells over 24 h revealed some reduction in metabolic activity at high concentrations (alamarBlue, Figure 3b). However, at the concentration (2.5 µM) required to achieve maximal inhibition of adenovirus replication, cell metabolism was 90% of the control (Figure 3b). No change in cell viability as assessed by trypan blue was observed over the full range of doses tested (0–40 µM). To gain greater insight into the basis for the reduction in virus replication by GPS491, we examined adenovirus early (E1A) and late (hexon) protein expression. Immunofluorescence analysis of hexon expression 24 h post infection (p.i.) revealed a reduction in the proportion of hexon-positive cells and extent of staining upon the addition of GPS491 (Figure 4a). Subsequent Western blot analysis of cell lysates at 8, 16, or 24 h p.i. determined that E1A expression was delayed upon GPS491 addition, being readily detected at 16 h p.i. rather than 8 h as in the control (Figure 4b). In contrast, although hexon expression was detectable as early as 16 h p.i. in the DMSO-treated samples, hexon expression was undetectable by Western blot up to 24 h p.i. in the presence of GPS491 (Figure 4b).

**Figure 5:**
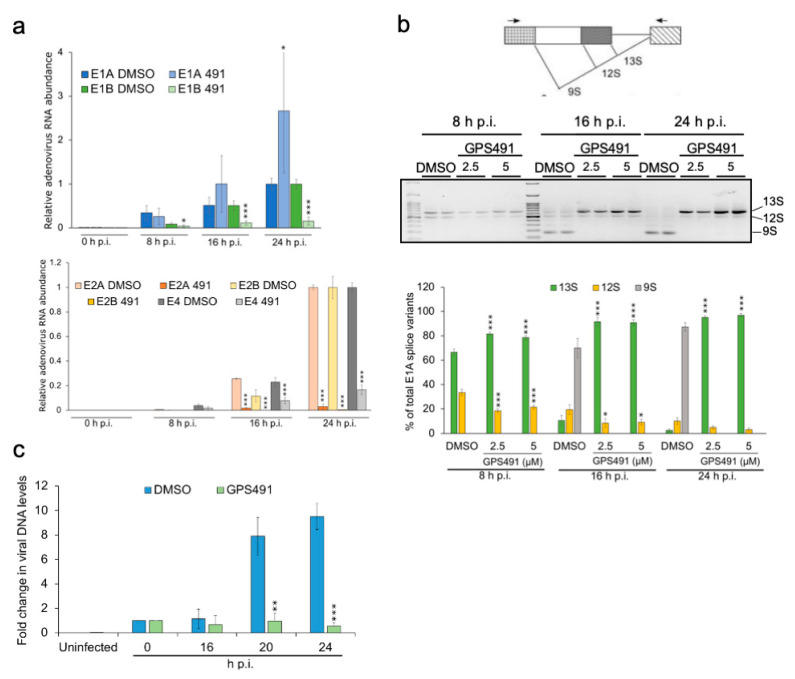
GPS491 alters adenovirus RNA expression/processing and inhibits adenovirus DNA amplification. A549 cells were infected with HAdV-C5 at an input MOI of 100 IU/cell for 1 h after which virus inoculum was removed and replaced with media containing DMSO or GPS491 (2.5 or 5 µM). (**a**) Total RNA was extracted at 8 h, 16 h, or 24 h after virus infection. After cDNA generation, RT-qPCR was performed using primers for E1A, E1B, E2A, E2B, and E4 RNAs (Supplementary Table S1). Values are expressed relative to RNA abundance observed 24 h p.i. in the presence of 1% DMSO. Shown are data from samples treated with 2.5 µM GPS491. (**b**) The effect of GPS491 on E1A RNA processing. Shown on the top is the schematic diagram of E1A RNA processing indicating the major E1A mRNA isoforms generated by alternative splicing. In the middle is a representative gel of the E1A RNA amplicons generated from cDNA. The graph on the bottom represents quantitation of amplicons generated across *n* > 3 independent assays. (**c**) At 16 h, 20 h, and 24 h p.i., total DNA was isolated from cells treated with DMSO or 2.5 µM GPS491 and levels of adenoviral DNA were determined by qPCR. Data are indicated as mean ± SD, * *p* ≤ 0.05, ** *p* ≤ 0.01, and *** *p* ≤ 0.001.

To assess whether the delayed expression of E1A upon GPS491 addition could be attributed to changes in viral RNA accumulation or splicing, both RT-qPCR and RT-PCR analyses were performed. Analysis of the kinetics of adenovirus early gene RNA accumulation revealed that, with the exception of E1A, the induction of multiple adenovirus early genes (E1B, E2A, E2B, E4) was significantly reduced in the presence of GPS491, with E2A and E2B RNAs being the most affected (Figure 5a). In contrast, E1A RNA abundance increased in the presence of GPS491 over 24 h. Given the disparity between the effect of GPS491 on adenovirus E1A protein and RNA accumulation, we asked whether some of the effects are due to changes in E1A RNA processing. The E1A transcript is the first viral RNA generated upon adenovirus infection and can be processed into multiple mRNAs by alternative splicing: the major variants are designated as 13S, 12S, and 9S (Figure 5b). However, the abundance of the different E1A RNA isoforms changes over the course of infection; 13S and 12S predominate at early times p.i., while 9S is the major E1A RNA at later times [24,25,26] (Figure 5b). As shown in Figure 5b, analysis of E1A RNAs over 24 h revealed a marked alteration in E1A RNA processing in the presence of GPS491. At 8 h p.i., 13S and 12S RNAs were the dominant isoforms in DMSO control and GPS491-treated samples, but GPS491 addition increased the relative abundance of 13S RNA. At later times p.i., while DMSO-treated cells shifted E1A RNA accumulation to 9S RNA, GPS491 treatment increased the predominance of 13S RNA, with this isoform representing >90% of all E1A RNAs by 16 h p.i. (Figure 5b).

While the changes in E1A RNA processing could explain the altered kinetics of E1A protein expression, the basis for the loss of hexon remained unclear. During adenovirus infection, transcription of the late genes is dependent upon viral DNA replication [27,28].

To determine whether the loss of hexon synthesis could be explained by an inhibition of viral DNA synthesis, qPCR was used to measure the relative abundance of the adenovirus genome in the presence of DMSO or GPS491. Consistent with the loss of late gene expression, GPS491 addition maintained viral DNA levels comparable to that observed immediately after virus addition (Figure 5c). Together, these observations indicate that GPS491 affects early events in adenoviral gene expression essential for viral genome replication.

### 3.3. GPS491 Alters Host SR Protein Abundance, Modification, and Function

**Figure 6:**
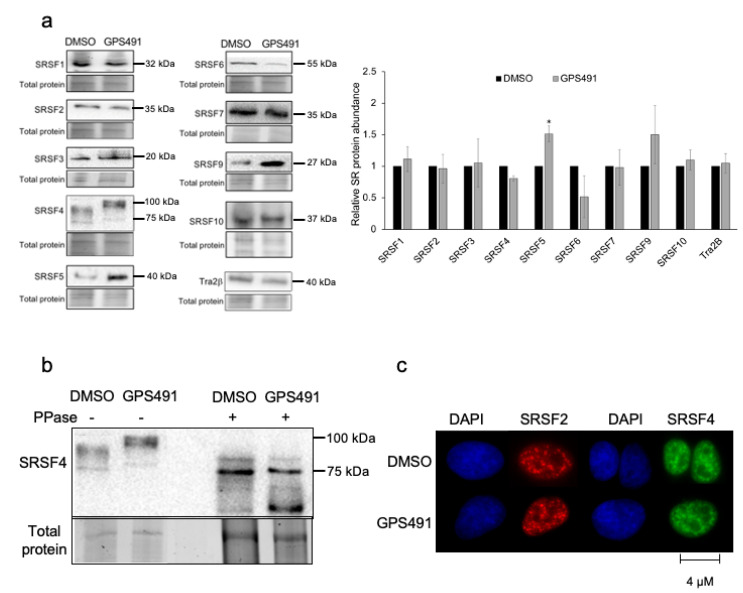
GPS491 alters expression and phosphorylation of selected SR proteins. HeLa rtTA HIV∆mls cells were treated with DMSO or GPS491 (2.5 µM) and induced with Dox. Twenty hours post induction, cells were harvested in RIPA buffer containing a phosphatase inhibitor cocktail and fractionated on 10% stain-free SDS-PAGE gels. (**a**) Representative Western blots for each SR protein are shown. At the right, a summary of Western blots from three independent assays measuring the effect of GPS491 on SR proteins levels relative to DMSO-treated cells. Asterisks (*) represent changes in expression level at *p* < 0.05. (**b**) Cell lysates were mock-treated or treated with alkaline phosphatase (PPase) prior to loading onto 10% stain-free gels. After transfer onto PVDF, blots were probed for SRSF4. Blots are representative of *n* > 3 independent assays. (**c**) Cells treated with DMSO or GPS491 were fixed and permeabilized followed by staining with antibodies to either SRSF2 or SRSF4. DAPI was used to stain for cell nuclei. Images were captured on Leica DMR Microscope at 63× oil immersion.

Given the shift in HIV-1 and adenovirus RNA processing/accumulation upon GPS491 addition, we examined whether some of the effects could be attributed to changes in abundance or modification of SR proteins, cellular factors with known roles in regulating RNA splicing as well as other facets of RNA processing [29,30,31]. Lysates from HeLa rtTA HIV∆mls cells treated with either DMSO or GPS491 for 24 h were fractionated on SDS-PAGE gels and individual SR proteins detected by Western blotting. Shown in Figure 6a are representative blots as well as a summary of the data from the three independent assays. Treatment with GPS491 selectively altered the abundance of several members of the SR protein family, increasing levels of both SRSF5 and SRSF9 by 1.5-fold, while reducing the abundance of SRSF6 by 50%, respectively. Limited changes in the levels of other SR/SR-related factors were observed. However, in addition to changes in SR protein abundance, we also noted a shift in SRSF4 migration consistent with an increase in its extent of phosphorylation (Figure 6b). To address whether this was indeed the case, cell extracts were treated with or without alkaline phosphatase prior to fractionation on SDS-PAGE and Western blotting. As indicated in Figure 6b, treatment with phosphatase increased SRSF4 mobility and eliminated the mobility difference observed between DMSO- and GPS491-treated samples, consistent with the altered mobility being due to increased phosphorylation. Given that SR hyperphosphorylation is also associated with changes in its subnuclear distribution (moving from nuclear speckles to more uniform nuclear staining [32,33,34,35]), we examined the effect of GPS491 treatment on both SRSF2 and SRSF4 subcellular distribution. Despite the change in SRSF4 phosphorylation upon GPS491 addition, SRSF4 and SRSF2 localization to nuclear speckles remained unaffected (Figure 6c).

**Figure 7:**
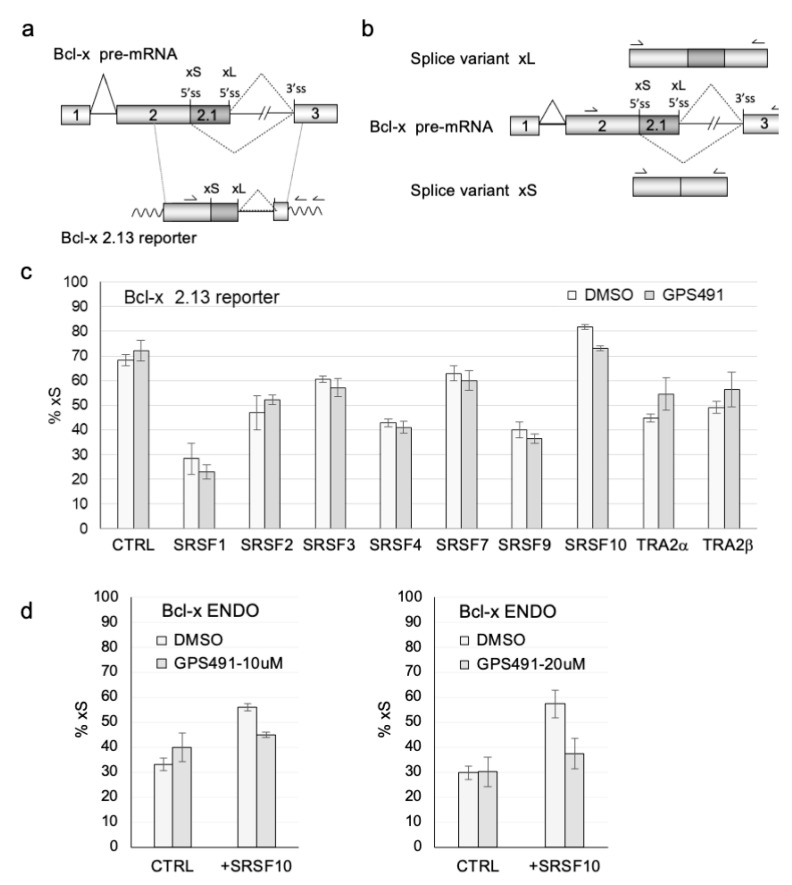
GPS491 alters the function of only a subset of SR proteins. (**a**) Map depicting the Bcl-x pre-mRNA and the Bcl-x minigene 2.13 as well as the position of the primers used for RT-PCR. (**b**) Map of the endogenous Bcl-x gene and spliced products Bcl-xS and Bcl-xL. Positions of primers for RT-PCR are indicated. (**c**) HEK 293 cells were transfected with Bcl-x reporter along with plasmids expressing the indicated SR proteins, followed by treatment with DMSO or 10 μM GPS491. After 48 h, total RNA was isolated and RT-PCR was performed. The relative abundance of the xL and xS products was determined following fractionation on PAGE gels. (**d**) Assays were repeated with overexpression of SRSF10 in the presence of DMSO and 10 or 20 µM GPS491, evaluating the effect on alternative splicing of the endogenous Bcl-x gene (Bcl-x ENDO). Representative gel images are provided in Supplementary Figure S3.

To evaluate the effect of GPS491 on SR function, we also examined whether the compound altered SR protein activity as measured by their modulation of Bcl-x RNA splicing. In this instance, cells were transfected with a Bcl-x reporter vector (Bcl-x 2.13, Figure 7a) along with control vector plasmids (CTRL, CMVmyc) or ones expressing individual SR proteins. As indicated in Figure 7c and Supplementary Figure S3a, plasmid-derived expression of most SR proteins in the absence of the compound decreased the use of the Bcl-xS 5′ss, only transfection of SRSF10 yielding the opposite effect. The addition of 10 µM GPS491 had a limited or no effect on the response to most SR proteins tested (Figure 7c), except for SRSF10, where it decreased the ability of SRSF10 to promote Bcl-xS 5′ss usage. As a confirmation of this effect, we examined the impact of GPS491 on endogenous Bcl-x splicing (Figure 7b) and noted that GPS491 also prevented the SRSF10-mediated switch towards more endogenous Bcl-xS RNA (Figure 7d and Supplementary Figure S3b). Given the changes in SR protein abundance, phosphorylation, and activity observed, we examined whether any of the changes were due to alteration of SR kinase expression. As shown in Supplementary Figure S4, tests in the JLat 10.6 cell line did not detect any significant alteration in CLK1-3 expression, but SRPK1 expression increased 2.5-fold upon GPS491 addition.

### 3.4. GPS491 Inhibits Coronavirus Replication

**Figure 8:**
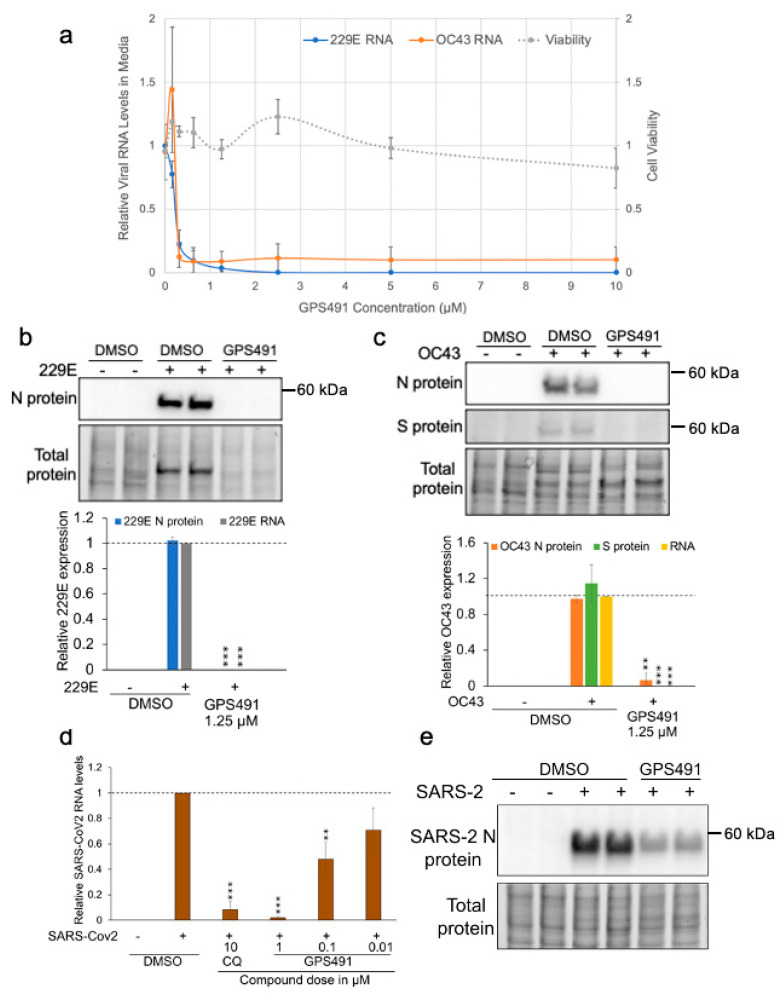
GPS491 inhibits replication of 229E, OC43, and SARS-CoV2 coronaviruses. (**a**) Huh7 cells were infected with either 229E or OC43 at an input MOI of 0.1 and 1, respectively, for 1 h. Virus inoculum was removed, cells were washed, and fresh media containing 1% DMSO or varying concentrations of GPS491 (from 0 µM to 10 µM) were added. Then, 2 days p.i (229E) or 4 days p.i. (OC43), media were harvested to quantitate viral genomic RNA levels by RT-qPCR assay. All the values are expressed relative to the values detected in virus-infected DMSO-treated samples. Effect of GPS491 on cell viability was assessed 2 days post compound addition using alamarBlue at the indicated doses of GPS491. Data are indicated as mean ± SD, ** *p* ≤ 0.01, and *** *p* ≤ 0.001. (**b**,**c**) Huh7 cells were infected with (**b**) 229E at an MOI of 0.03 or (**c**) OC43 at an MOI of 0.3 for 1 h after which the virus inoculum was removed, cells were washed with 1× PBS, and media containing 1% DMSO or 1.25 µM GPS491 were added. Cells and media were harvested 2 days (229E) or 4 days (OC43) p.i., and the levels of viral proteins (N, S) were determined by Western blot and virus production was assessed by RT-qPCR of the media (RNA). Shown are representative Western blots and their respective quantitation indicating the effects of GPS491 following infection with 229E and OC43, respectively. Band intensity was quantified relative to virus-infected control and normalized to total protein load (stain-free gels). Data are indicated as mean ± SD and generated from *n* = 3 independent assays, each performed in duplicate. (**d**,**e**) Huh7 cells were infected with SARS-CoV2 at an MOI of 1 for 1 h. Virus inoculum was removed, and fresh media containing DMSO, chloroquine (CQ, 10 µM), or indicated doses of GPS491 were added. Two days post infection, (**d**) media were harvested, and levels of virion production determined by RT-qPCR or (**e**) lysates from cells treated with 1% DMSO or 0.3 µM GPS491 were blotted to measure expression of viral N protein. Results shown were generated from *n* = 3 independent assays.

To explore whether GPS491 could impact the replication of other viruses, we tested its effect against coronaviruses. As positive strand RNA viruses, the replication cycle of coronaviruses is restricted to the cell cytoplasm and has no direct dependence on cellular RNA splicing [36,37,38,39]. However, GPS491 addition 1 h p.i. resulted in a dose-dependent reduction (EC_50_ ~250 nM/CC_50_ >10 µM in Huh7 cells, Figure 8a) of coronavirus replication as evidenced by loss of 229E and OC43 viral RNA accumulation in the media (indicative of viral particle formation). Subsequent analyses (Figure 8b,c) determined that GPS491 addition drastically reduced 229E and OC43 nucleocapsid (N) and spike (S) protein expression in cell lysates. Success against the seasonal coronaviruses led us to test whether GPS491 had a similar capacity to suppress replication of SARS-CoV2. As shown in Figure 8d,e, GPS491 reduced SARS-CoV2 genome replication/expression in a dose-dependent manner (EC_50_ ~100 nM) in Huh7 cells as evidenced by loss of viral genomic RNA accumulation in the media and nucleocapsid expression in the cell.

**Figure 9:**
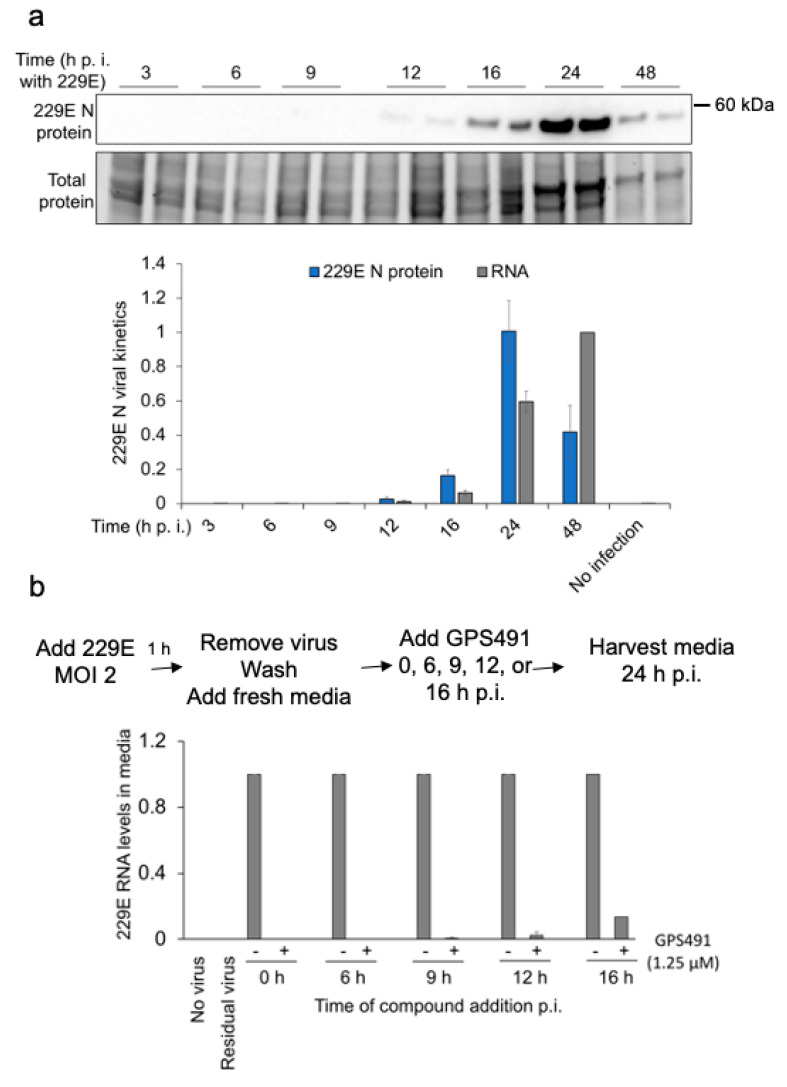
GPS491 inhibits 229E virus replication post entry. (**a**) Kinetics of 229E coronavirus N protein expression and virus release. Huh7 cells were infected at an input MOI of 2. After incubation of cells for 1 h, cells were washed to remove residual virus and fresh media were added. At indicated times post infection (p.i.), cells and media were harvested. Cell extracts were analyzed by Western blot for N protein expression, while RTqPCR was used to monitor levels of viral RNA in the media. Shown is a representative Western blot as well as a graphical summary of the quantitative results obtained from *n* = 3 independent assays. (**b**) Addition of GPS491 at late stages of 229E replication suppresses virus growth. Huh7 cells were infected at an input MOI of 2. After incubation of cells for 1 h, cells were washed to remove residual virus and fresh media were added. At indicated times post infection (p.i.), DMSO or GPS491 was added to cultures at a final concentration of 1.25 µM and incubation continued. At 24 h post-virus infection, media were harvested and the abundance of viral RNA in media was determined by RTqPCR.

To gain a greater understanding of the stage of coronavirus replication affected by GPS491, we first studied 229E viral replication kinetics by measuring nucleocapsid expression in cell lysates and virion RNA release in media at different times p.i. (at an input MOI of 2). Analysis of 229E kinetics of nucleocapsid expression determined that viral nucleocapsid was first detectable at 12 h p.i, gradually increasing to a maximum at 24 h p.i. (Figure 9a). Accumulation of viral genomic RNA in the media, an indicator of virion assembly and release, was first evident at 16 h p.i., reaching a maximum at 48 h p.i. (Figure 9a). We next performed time of addition assays, in which the compound was added at different time points (0, 6, 9, 12, or 16 h p.i.) to determine whether the compound was effective at all stages of the virus replication cycle. These analyses (Figure 9b) revealed that delaying GPS491 addition to cells had minimal effect on its ability to inhibit virus replication, with addition at 12 or 16 h p.i. being almost as effective as 0 h p.i. in reducing the accumulation of viral genomic RNA in the media 24 h p.i.

## 4. Discussion

Due to the high variability in polymerase/protease sequences/structures within and among different viruses, compounds targeting these proteins may show limited activity against unrelated viruses. Furthermore, the high error rate of the viral polymerases provides the viruses with an ability to rapidly evolve resistant viral variants once the drug is applied. However, the dependence of all viruses on cellular processes for their replication suggests that host factor manipulation may provide a robust target for therapies with the additional benefit that such treatments may affect multiple viruses dependent on the same process. One example is alternative RNA splicing, which multiple mammalian viruses exploit to greatly expand their coding capacity without having to increase their genome size. Examples include influenza, retroviruses, adenoviruses, polyoma, and papilloma viruses [24,40,41,42,43,44,45,46,47]. RNA splicing is a multistep process involving the assembly of a ~1.8 megadalton RNA–protein complex known as the spliceosome [48] and is a key point of gene expression regulation for >90% of human RNA polymerase II transcripts that undergo alternative splicing [49]. Splice site selection is governed by the action of multiple splicing regulatory factors [50,51]. For many viruses, balancing the extent of alternative splicing is essential for virus replication, since altering the proportion of individual viral mRNAs leads to changes in viral protein expression and a severe reduction in virion yield [6,7,17,19,52,53,54]. Consequently, altering the relative activity of key cellular factors regulating particular stages of RNA processing could be the basis of a broad-spectrum antiviral in contrast to existing treatment paradigms that target virus-encoded enzymes. Such host-targeted therapies (HTT) could also provide a higher barrier to the emergence of resistance [4,5,55], particularly in the case of splicing, where the virus would need to alter its interaction with multiple components of the splicing apparatus to establish a new, productive equilibrium. In support of this hypothesis, studies with the anti-HIV-1 compound ABX464 have shown a reduction in HIV-1 replication in both humanized mice and humans without the appearance of resistant HIV-1 strains [56,57,58]. Other studies have identified compounds (IDC16, 1C8, 791, 5342191, 5350150) [7,9,19,52,56,59,60] that can dramatically reduce HIV-1 gene expression through alterations in viral RNA abundance with limited effects on host RNA accumulation, indicating a greater sensitivity of viral RNA to minor perturbations in the host RNA processing machinery.

Although the current data do not identify the target host factor, which mediates GPS491′s effect on the viruses studied, the compound’s ability to inhibit the replication of multiple different viruses demonstrates its potential as a broad-spectrum antiviral. In the context of HIV-1, GPS491 inhibited multiple HIV-1 variants, including those resistant to current therapeutics. GPS491′s reduction of HIV-1 protein expression in cell lines (HeLa rtTA HIV∆mls, J-Lat 10.6) containing integrated HIV-1 proviruses indicates that it acts by affecting post-integration events not targeted by current drugs. Consistent with the observed loss of Gag and Env, GPS491 treatment significantly reduced the abundance of their corresponding RNAs to a greater extent than seen with 5350150 [7]. Levels of HIV-1 MS RNA were slightly elevated, indicating that GPS491 has limited effect on transcription from the viral promoter but alters the balance in the processing of the primary transcript to favor the formation of the fully spliced form of the viral RNA. Similar to 5350150, GPS491 treatment induced changes in splice site usage within the MS RNAs to favor Tat1 RNA [7], but also reduced Rev1/2 RNA, with the resulting reduced level of Rev protein possibly affecting viral US and SS RNA accumulation in the cytoplasm [61,62]. Unlike 5350150, GPS491 treatment significantly reduced Tat protein levels. The ability of proteasome inhibition (through addition of MG132) to restore Tat p16 expression suggests that GPS491 reduces either the translation of MS RNA encoding Tat or the stability of the Tat protein.

In the case of adenovirus, treatment of cells with GPS491 did not block genome delivery to the nucleus as evidenced by the presence of E1A RNA and protein. However, the compound affects a very early phase of virus gene expression as E1A RNA accumulation was enhanced while the expression of other early genes was significantly reduced, indicating that the regulatory cascades driving their expression were interrupted by GPS491. One example is the loss of E2A and E2B, whose transcription is dependent upon the induction of the host E2F transcription factor mediated by the combined action of adenovirus E1A and E4 proteins [63,64]). Other effects are possibly due to the altered synthesis of E1A isoforms produced by alternative splicing of E1A RNA. Addition of GPS491 strongly favoured the accumulation of the 13S E1A RNA. The failure of E1A RNA processing to transition (from predominantly 13S/12S early to 9S late) in the presence of GPS491 may be attributable to the loss of E4 as the E4-orf4 protein interacts with the cellular PP2A phosphatase to induce hypo-phosphorylation of cellular SR proteins [24,65,66]. With GPS491 affecting expression of so many of the early proteins critical for viral DNA replication, the observed lack of viral DNA amplification is expected. Similarly, the loss of late viral gene expression (i.e., hexon) is anticipated given its dependence on viral DNA replication [27,28].

The changes in HIV-1 and adenovirus RNA accumulation induced by GPS491 suggested that it affected the cellular RNA processing machinery. Consistent with this hypothesis, we did observe changes in SR protein abundance, modification, and function upon GPS491 addition, in particular a reduction in SRSF10 function, which plays a critical role in HIV-1 and hepatitis B replication [52,67]. Suppression of virus gene expression upon GPS491 addition occurred with limited changes in abundance of several cellular SR kinases, with only SRPK1 levels being affected (in contrast to a known SR kinase inhibitor TG003 [68], Supplementary Figure S4). Both overexpression and depletion studies have highlighted several members of the SR protein family that play pivotal roles in regulating HIV-1 gene expression and virus replication [69,70,71,72,73,74,75,76]. Studies have highlighted the effects of both SRSF5 and SRSF4 in regulating different facets of HIV-1 gene expression. In the case of SRSF5, in vitro studies indicated that this factor weakly modulates the use of particular splice sites within HIV-1 RNA [70,77]. Subsequent in cell SRSF5 depletion and overexpression experiments determined that, while SRSF5 depletion had limited effect on HIV-1 RNA accumulation, its overexpression did induce changes in splice site selection and reduced expression of HIV-1 Gag [70,71,78]. Studies have also highlighted a role for SRSF5 in facilitating the translation of HIV-1 Gag RNA [79,80], but this effect was only documented in the context of murine cell lines. Less information is available regarding the effects of SRSF4 modulation on HIV-1 RNA processing. SRSF4 overexpression increases HIV-1 Gag expression and alters the usage of specific splice sites within the viral RNA [81,82], while its depletion results in the selective loss of RNAs encoding Vpr [78]. While SR protein phosphorylation is known to affect the activity and localization of the protein [33,83,84,85]), it is unclear how the changes in SR phosphorylation but not localization induced by GPS491 impact function. The effects of GPS491 on SR protein function (Figure 7c,d) were limited to SRSF10, an unexpected result in light of the absence of changes in SRSF10 abundance or apparent phosphorylation upon GPS491 addition (Figure 6). Previous studies [52] have demonstrated a significant role for SRSF10 in regulating HIV-1 replication, with its depletion or altered phosphorylation resulting in marked alterations in HIV-1 expression and RNA accumulation. While the splicing modulator 1C8 altered SRSF10 activity and inhibited HIV-1 replication through alterations in viral RNA accumulation, its effects were distinct from that of GPS491 [52]. Unlike GPS491, 1C8 addition reduced accumulation of all HIV-1 mRNAs, with low doses (1 µM) preferentially reducing levels of spliced (SS and MS) viral RNAs. Consequently, while both 1C8 and GPS491 affect SRSF10 function, their distinct effects on viral RNA accumulation suggest that their mechanisms of action are different. Evidence demonstrating a role for individual SR proteins in the control of adenovirus replication is more limited. Studies have determined that SRSF1 overexpression blocks adenoviral DNA replication and the cytoplasmic accumulation of late viral mRNAs [86], while modulation of SRSF4 expression alters E1A RNA alternative splicing to favor the 12S form [87].

SR proteins also play essential roles in replication of other viruses (i.e., hepatitis B [67,88,89]) not known to require splicing of their RNAs. Studies of viral RNA interactomes of multiple cytoplasmic RNA viruses have revealed a common set of host-interacting proteins [90,91] and identified roles for SR proteins and the cellular kinases (SRPK1,2) that modify SR protein activity in the replication of several RNA viruses (polio, hepatitis C, Sindbis, SARS-CoV-1/2) [82,92,93,94]. Although first identified for their role in RNA splicing [29,51,95,96,97,98,99,100], SR proteins can modulate several other stages of RNA metabolism critical for virus replication including transcription initiation/elongation, mRNA polyadenylation, export to the cytoplasm, and translation [30,51,79,80,101,102,103]. Consequently, modulating SR protein activity could alter their effect at any of these stages of gene expression.

The ability of GPS491 to inhibit multiple coronaviruses (229E, OC43, SARS-CoV-2) was unexpected given that replication of these viruses occurs in the cytoplasm [39,104], independent of the cellular nuclear RNA processing machinery involved in HIV-1 or adenovirus expression. Characterization of host proteins interacting with SARS-CoV2 RNA has revealed the association of several hnRNPs and SR proteins (including SRSF10), suggesting a possible role in regulating coronavirus replication [90,91]. Consistent with this hypothesis, parallel CRISPR screens determined that the depletion of a subset of SR proteins can negatively impact coronavirus replication [105]. In addition, the nucleocapsid (N) protein of multiple coronaviruses contains a highly conserved region of arginine-serine repeats (similar to those in SR proteins) whose phosphorylation by host kinases (SRPK1, GSK-3) regulates the protein oligomerization required for virus replication [94,106,107,108]. Inhibition of SRPK1 or GSK-3 function dramatically reduces coronavirus replication [94,108]. GPS491′s ability to block coronavirus virus particle formation when added 1 or 16 h after virus infection indicates that it is a potent post-entry inhibitor of virus replication, able to arrest virus replication even after the onset of virus structural protein production. Whether GPS491 acts directly to affect important steps in viral RNA/protein synthesis or indirectly through modulation of abundance or activity of a key host factor(s) remains to be determined. In support of the latter possibility, studies of SARS-CoV2 have revealed a requirement for RNA splicing as treatment of cells with pladienolide B [109], an inhibitor of the spliceosome factor SF3B1, reduced virus replication.

Together, the observations presented in this report demonstrate that GPS491 is a potential broad-spectrum, host-directed, antiviral agent displaying potent activity against three families of unrelated viruses, from retroviruses to coronaviruses. We have shown that the compound effects are associated with the alteration of some host factors. In addition to the broad antiviral spectrum of GPS491, we also documented the activity of the compound against drug-resistant strains of HIV-1, suggesting its utility as a salvage therapy in patients who fail current virus-directed treatments. Additional studies to define the mechanism by which GPS491 alters the cell to render it unable to support virus replication will prove invaluable in refining the strategy to enhance antiviral potency while limiting the effect on the cell and the host.

## Figures and Tables

**Table 1:** Effect of GPS491 on replication of multiple mammalian viruses.

Virus	Strain	EC_50_	CC_50_
HIV-1 Assayed in CEM-GXR cell line	Clade A	176 nM	12,052 nM
	Clade B	248 nM	12,052 nM
	NL43 E0043 RTI	235 nM	12,052 nM
	NL43 2918 PI	230 nM	12,052 nM
	NL43 11845 INI	203 nM	12,052 nM
	IIIB MVC res	216 nM	12,052 nM
HIV-1 Assayed in PBMCs	HIV-1 Bal (average of 3 donors)	248 nM	2,509 nM
	HIV-1 IIIB (average of 3 donors)	738 nM	2,509 nM
Adenovirus Assayed in A549 cell line	HAdV-C5	1000 nM	>40,000 nM
Coronavirus Assayed in Huh7 cell line	229E	250 nM	>10,000 nM
	OC43	250 nM	>10,000 nM
	SARS-CoV2	100 nM	>10,000 nM

See Figure S1 for data of the effect of GPS491 on HIV-1 replication.

## Supplementary Materials

The following are available online at https://www.mdpi.com/article/10.3390/v14010060/s1, Table S1: RT-qPCR primer sequences, Figure S1: Anti-HIV-1 activity of GPS491 against wild-type clade A and B and drug-resistant viruses Figure S2: GPS491 reduces HIV-1 Gag expression in J-Lat 10.6 cells, Figure S3: GPS491 modifies the function of SRSF10, Figure S4: Effect of GPS491 on SR kinase expression.

## Abbreviations

ATCC: American Type Culture Collection, cART: combination antiretroviral therapy, cDNA: complementary DNA, DMEM: Dulbecco’s Modified Eagle Medium, DMSO: dimethyl sulfoxide, Dox: doxycycline, FBS: fetal bovine serum, HAdV-C5: human adenovirus serotype 5,HEK 293: Human Embryonic Kidney 293, HTT: host-targeted therapeutics, IMDM: Iscove’s Modified Dulbecco’s Medium, MEM: minimum essential medium, MOI: multiplicity of infection, MS: multiply spliced, N: nucleocapsid, PBMCs: peripheral blood mononuclear cells, p.i.: post infection, P/S: penicillin/streptomycin, RPMI 1640: Roswell Park Memorial Institute 1640, RT: reverse transcription, S: spike, SR: serine-arginine-rich splicing factor, SS: singly spliced, US: unspliced.
