# Supplementary material for: The Thiazole-5-Carboxamide GPS491 Inhibits HIV-1, Adenovirus, and Coronavirus Replication by Altering RNA Processing/Accumulation
Source: Viruses. 2021 Dec 30;14(1):60. doi: 10.3390/v14010060 (PMC8779516; doi:10.3390/v14010060)
Supplement: Supplementary file 1 [file viruses-14-00060-s001.zip › viruses-1505198-supplementary.pdf]

viruses

MDPI

Supplementary Materials

The Thiazole-5-Carboxamide GPS491 Inhibits HIV-1, Adenovirus, and Coronavirus Replication by Altering RNA Processing/Accumulation

Table S1. RT-qPCR Primer Sequences.

Virus	Target gene	Forward primer	Reverse primer
Coronaviruses	229E	5′-CGCAAGAATTCAGAACCAGAG-3′	5′-GGCAGTCAGGTTCTTCAACAA-3′
	OC43	5′-GCTCAGGAAGGTCTGCTCC-3′	5′-TCCTGCACTAGAGGCTCTGC-3′
	SARS-CoV2	5′GACCCCAAAATCAGCGAAAT-3′	5′-TCTGGTTACTGCCAGTTGAATCTG-3′
Adenovirus	E1A	5′-GGAATACGGGGGACCCAGA-3′	5′-ATTTTAGGACGGCGGGTAGG-3′
	E1B	5′-ACATACTGACCCGCTGTTCC-3′	5′-AAACACCCCGTTCAGGTTCA-3′
	E2A	5′-CGGTCTGGGCGTTAGGATAC-3′	5′-TGCAGATCTCCAACACCGAC-3′
	E2B	5′-TTGTTGTGTAGGTACTCCGCC-3′	5′-CCTTGCGACTGTGACTGGTT-3′
	E4	5′-AGGCGCTGTATCCAAAGCTC-3′	5′-TCCAGCGTGTTTATGAGGGG-3′
	TBP	5′-GATGCCTTATGGCACTGGAC-3′	5′-GCCTTTGTTGCTCTTCCAAA-3′
HIV-1	US	5′-GACGCTCTCGCACCCATCTC-3′	5′-CTGAAGCGCGCACGGCAA-3′
	SS	5′- GGCGGCGACTGGAAGAAGC-3′	5′- CTATGATTACTATGGACCACAC-3′
	MS	5′-GACTCATCAAGTTTCTCTATCAAA-3′	5′-AGTCTCTCAAGCGGTGGT-3′
	ß-actin	5′-TGACGTGGACATCCGCAAAG-3′	5′-CTGGAAGGTGGACAGCGAGG-3′
	RT-PCR MS RNA	5′-CTGAGCCTGGGAGCTCTCTGGC-3′	5′-CCGCAGATCGTCCCAGATAAG-3′

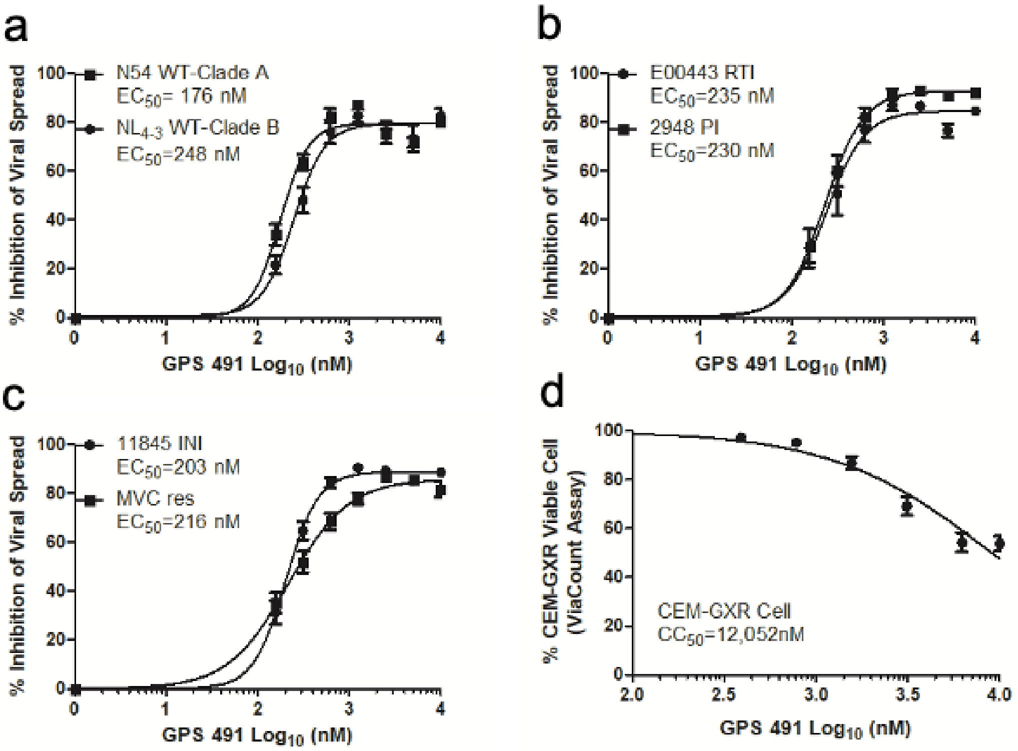

Figure S1. Anti-HIV-1 activity of GPS491 against wild-type clade A, B, and drug-resistant viruses. (**a**) N54 (clade A) and HIV-1$_{NL4-3}$ (clade B), (**b**) (N)NRTI-resistant isolate and PI-resistant isolate, (**c**) INI-resistant isolate and maraviroc-resistant R5 strain. In the presence of increasing GPS491 concentrations, HIV-1 infectivity on CEM-GXR cells was assessed by measuring GFP positive cells 3 days after infection. The inhibition curves were then fitted by nonlinear regression with GraphPad Prism software, allowing EC50 calculation. Results represent the average of three independent determinations. (**d**) Evaluation of GPS491 on CEM-GXR cell viability in the Guava ViaCount assay. Cells were analyzed after 72 h incubation with compound GPS491 in concentrations ranging between 0.2 and 10 µM. Results are expressed as the percentage (%) of viable cells ± SEM of three independent experiments.

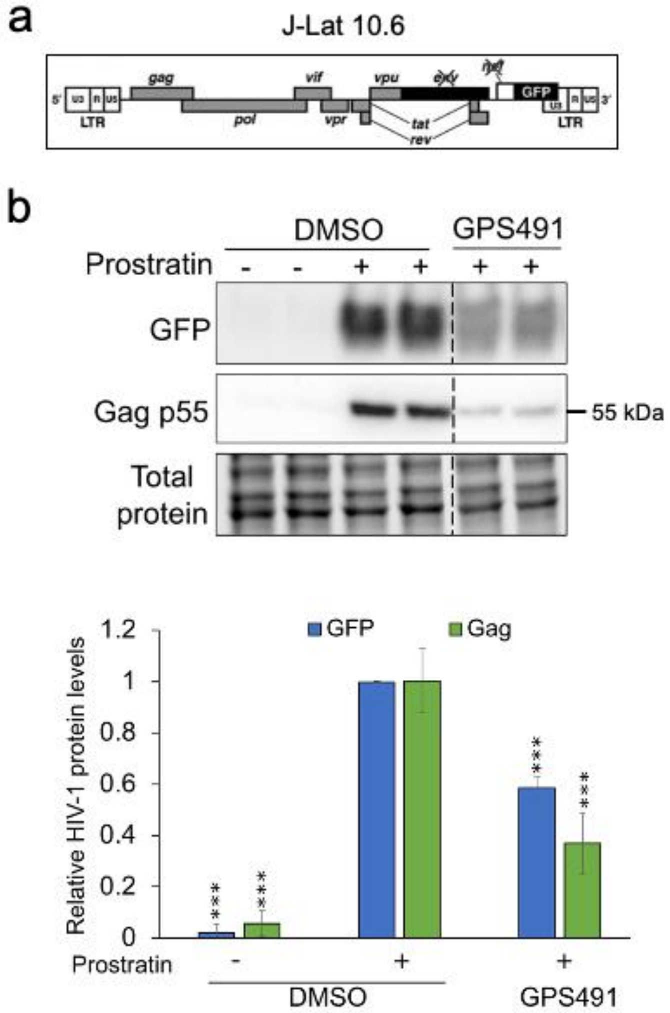

Figure S2. GPS491 reduces HIV-1 Gag expression in J-Lat 10.6 cells. (**a**) provirus in J-Lat 10.6 cells. The construct has a substitution of GFP for Nef. The genome generates replication defective virions due to a frameshift in Env gene. (**b**) J-Lat 10.6 cells were treated with either 1% DMSO or GPS491 (2 or 1 µM) and provirus expression induced by addition of 1 µg/mL of prostratin. After 24 h, cells were harvested, and lysates fractionated on 10% stain-free SDS PAGE gels. Gels were imaged to detect GFP signal or total protein (stain-free). After transfer of the gels, blots were probed to detect HIV-1 Gag. Shown are the representative blots. Dotted vertical lines on the blots represent cropping of lanes on the same representative blot to show DMSO control lanes adjacent to compound treatment lanes. Below, quantitation of western blots representing n>3 independent assays. Values are normalized for total protein and expressed relative to DMSO treated and induced controls. Data are indicated as mean ± SD, ***$p \leq 0.001$.

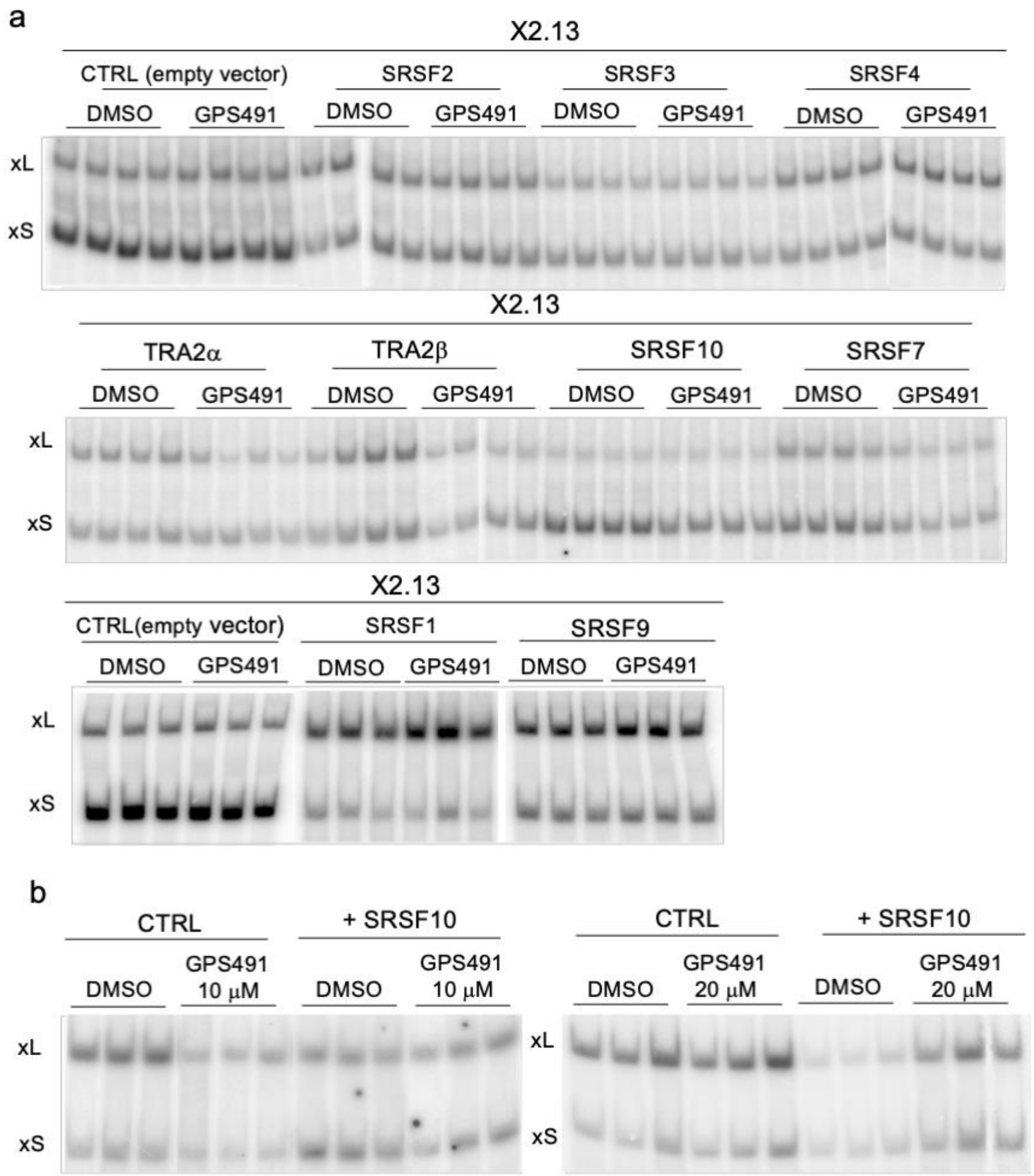

Figure S3. GPS491 modifies the function of SRSF10. (**a**) HEK 293 cells were transfected with *Bcl-x* reporter along with plasmids expressing the indicated SR proteins and treated with DMSO or 10 μM GPS491. After 48 h, total RNA was harvested, RT-PCR performed, and the relative abundance of xL and xS products from the reporter determined after fractionation on PAGE gels. Shown are the representative gels. (**b**) HEK 293 cells were transfected with plasmid SRSF10 and then treated with DMSO or GPS491 (10 or 20 μM). After 48 h, total RNA was harvested, RT-PCR performed, and relative abundance of endogenous xL and xS products determined after fractionation on PAGE gels. Representative gels are provided.

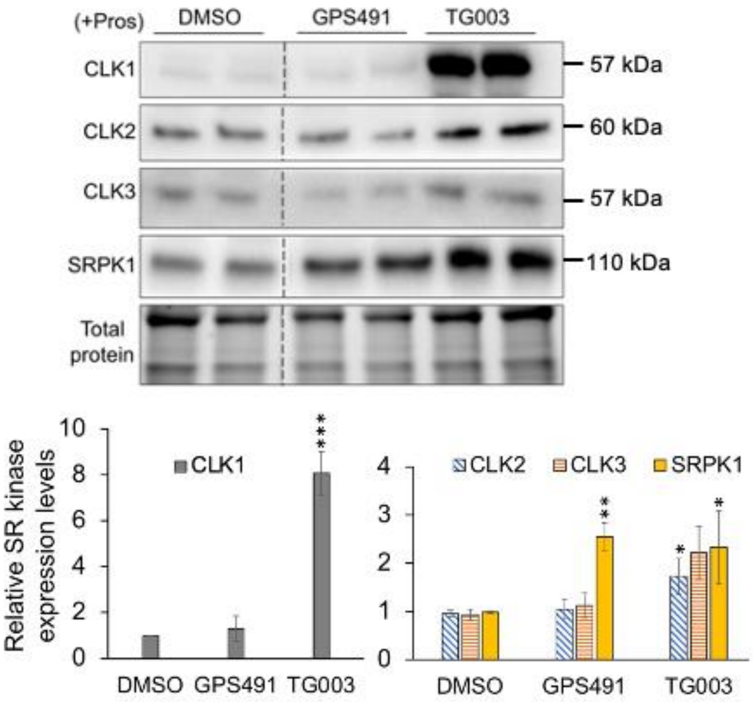

Figure S4. Effect of GPS491 on SR kinase expression. HIV-1 expression was induced by addition of prostratin (1 µg/mL) to JLat 10.6 cells then immediately treated with DMSO, GPS491 (1.25 µM), or the CLK inhibitor TG003 (10 µM). After 24 h, cell lysates were prepared, fractionated on SDS-PAGE gels, and proteins transferred onto PVDF. Blots were probed to detect CLK1, CLK2, CLK3, or SRPK1. Shown is a representative western blot as well as a quantitative summary of results from $n>3$ assays. Dotted vertical lines on the blots represent cropping of lanes on the same blot to show DMSO control lanes adjacent to compound treatment lanes. Stain-free gels (Bio-Rad) were used to normalize for total protein. Data are indicated as mean ± SD, *$p \leq 0.05$, **$p \leq 0.01$, and ***$p \leq 0.001$.
